# 16S rRNA Gene Sequence-Based Identification of Bacteria in Automatically Incubated Blood Culture Materials from Tropical Sub-Saharan Africa

**DOI:** 10.1371/journal.pone.0135923

**Published:** 2015-08-13

**Authors:** Hagen Frickmann, Denise Dekker, Norbert Georg Schwarz, Andreas Hahn, Kennedy Boahen, Nimako Sarpong, Yaw Adu-Sarkodie, Eva Halbgewachs, Florian Marks, Vera von Kalckreuth, Sven Poppert, Ulrike Loderstaedt, Jürgen May, Ralf Matthias Hagen

**Affiliations:** 1 Department of Tropical Medicine at the Bernhard Nocht Institute, German Armed Forces Hospital of Hamburg, Hamburg, Germany; 2 Institute for Medical Microbiology, Virology and Hygiene, University Hospital Rostock, Rostock, Germany; 3 Bernhard Nocht Institute for Tropical Medicine, Hamburg, Germany; 4 Kumasi Centre for Collaborative Research, Kumasi, Ghana; 5 Kwame Nkrumah University of Science and Technology, Kumasi, Ghana; 6 Laboratory Department 1, Central Institute of the German Armed Forces Medical Services Kiel, External site Berlin, Berlin, Germany; 7 International Vaccine Institute (IVI), Seoul, Korea; 8 Department of Medical Microbiology at the University Medical Centre Gießen, Justus-Liebig University Gießen, Gießen, Germany; 9 Department of Clinical Chemistry, University Medical Center Goettingen, Goettingen, Germany; Charité, Campus Benjamin Franklin, GERMANY

## Abstract

**Background:**

The quality of microbiological diagnostic procedures depends on pre-analytic conditions. We compared the results of 16S rRNA gene PCR and sequencing from automatically incubated blood culture materials from tropical Ghana with the results of cultural growth after automated incubation.

**Methods:**

Real-time 16S rRNA gene PCR and subsequent sequencing were applied to 1500 retained blood culture samples of Ghanaian patients admitted to a hospital with an unknown febrile illness after enrichment by automated culture.

**Results:**

Out of all 1500 samples, 191 were culture-positive and 98 isolates were considered etiologically relevant. Out of the 191 culture-positive samples, 16S rRNA gene PCR and sequencing led to concordant results in 65 cases at species level and an additional 62 cases at genus level. PCR was positive in further 360 out of 1309 culture-negative samples, sequencing results of which suggested etiologically relevant pathogen detections in 62 instances, detections of uncertain relevance in 50 instances, and DNA contamination due to sample preparation in 248 instances. In two instances, PCR failed to detect contaminants from the skin flora that were culturally detectable. Pre-analytical errors caused many Enterobacteriaceae to be missed by culture.

**Conclusions:**

Potentially correctable pre-analytical conditions and not the fastidious nature of the bacteria caused most of the discrepancies. Although 16S rRNA gene PCR and sequencing in addition to culture led to an increase in detections of presumably etiologically relevant blood culture pathogens, the application of this procedure to samples from the tropics was hampered by a high contamination rate. Careful interpretation of diagnostic results is required.

## Introduction

The distribution of bacterial infectious agents in blood culture materials of sepsis patients in tropical African or Asian countries [1–4] differs considerably from the situation in Western industrialized countries [5–7]. Coagulase-negative staphylococci are most frequently isolated from blood culture but can–with few exceptions–be considered as contaminants during sample acquisition. The major causative agents in the Western world comprise Gram-positive *Staphylococcus aureus*, *Enterococcus* spp., *Streptococcus* spp., Gram-negative *Escherichia coli*, *Enterobacter* spp., *Proteus mirabilis*, *Klebsiella* spp., *Pseudomonas aeruginosa*, and–in increasing proportions due to intensive care treatment–*Candida* spp. [5–7]. In Africa, *Salmonella enterica*, *Mycobacterium tuberculosis*, and *Streptococcus pneumoniae* are the predominant species isolated from bloodstream infections, followed by *S*. *aureus* and *E*. *coli* [2]. In South and South East Asia, *Salmonella* Typhi dominates, followed by *S*. *aureus*, *E*. *coli*, and other Gram-negative rod-shaped bacteria, while *S*. *pneumoniae* and *H*. *influenzae* are common in unvaccinated children with sepsis [3].

Routine microbiological diagnosis of bloodstream infections remains challenging in resource-limited areas. In a recent multiplex real-time PCR-based study in rural Ghana, we showed that even automated incubation missed about one-third of *Salmonella* spp.-induced bacteremia [8]. Our data are in line with previous publications suggesting a higher sensitivity of *Salmonella* PCR compared with cultural growth in the conditions encountered in tropical Africa [9, 10].

Considering this, we expected an even higher risk of missing bacteria that are not robust by automated incubation, resulting in a potential underestimation of their incidence in bloodstream infections. As has been known for decades, potentially relevant species such as *S*. *pneumoniae* and *H*. *influenzae* are particularly easily killed by environmental influences [11–15].

Pan-bacterial PCRs target evolutionarily highly conserved genetic elements, e.g., bacterial 16S or 23S rRNA genes [16]. Such PCRs with subsequent sequence analysis are well-established techniques for the identification of bacterial pathogens [17], allowing for the detection of a broad range of strains [18].

Various protocols of 16S rRNA gene-based broad-range PCRs for the diagnosis of bloodstream infections have been proposed [19]. PCR of short fragments shows a higher sensitivity than PCR of longer fragments of the 16S rRNA gene, while longer fragments provide better discrimination in sequence analysis [20]. Here we used two 16S rRNA gene PCRs, one intermediate-fragment PCR and one short-fragment PCR, with consecutive sequencing on 1500 residual volumes of automatically incubated blood culture specimens from a Ghanaian blood culture study after a locally affordable, centrifugation-based enrichment of bacterial DNA [8].

In this study, we aimed to estimate whether 16S rRNA gene-based (pan-bacterial) PCR and sequencing from automatically incubated blood culture materials in addition to cultural growth can lead to more reliable data for future epidemiological studies, particularly addressing bacteria that are easily killed by environmental influences such as *S*. *pneumoniae* or *H*. *influenzae*, which might have become autolytic prior to analysis through suboptimal conditions in the tropics, e.g., storage in tropical temperatures.

We analyzed the extent to which 16S rRNA gene sequencing leads to additional detections of blood culture pathogens in automatically incubated blood culture bottles in addition to cultural growth, and assessed the concordance of observed cultural growth with the 16S rRNA gene sequencing results. Finally, the potential suitability of 16S rRNA gene sequencing for future diagnostic purposes in the tropical setting is discussed in the light of decreasing prices for Sanger sequencing.

## Materials and Methods

### Analyzed specimens

Residual material of 1500 automatically incubated blood culture specimens (BACTEC Plus Aerobic F Medium/Peds Plus Medium, BD, Heidelberg, Germany), a subset of a recently described [8] sample collection from a blood culture study in rural Agogo, Ashanti Region, Ghana [4, 8, 21, 22], was included in the analysis. The epidemiological study from which the materials were taken has been described in detail recently [8]. In summary, within a study period of 2 years beginning in 2007, bottles with BD BACTEC Plus Aerobic/F Medium for adults and BD BACTEC Peds Plus Medium for children (BD, Heidelberg, Germany) were inoculated exactly as described in the manufacturer’s instructions with blood from patients with clinical signs of sepsis [8] in the Presbyterian Hospital of Agogo, Ghana. Bottles with BD BACTEC Plus Aerobic/F Medium were inoculated with 10 ml blood and bottles with BD BACTEC Peds Plus Medium were inoculated with 3 ml blood. Only one bottle per patient could be inoculated for organizational reasons. All suspected sepsis patients were included without any exclusion criteria. Inoculated blood culture bottles were stored at 20–30°C and transported at around 30°C in an air-conditioned car prior to incubation for 5 days in a BACTEC 9050 automated incubator (BD) at the Kumasi Centre for Collaborative Research, Kumasi, Ghana. A combined storage and transport time <24 hours was aimed for. After 5 days of automatic incubation of the inoculated blood culture bottles in the BACTEC 9050 automated incubator or when a culture was detected positive, 1 ml of the blood culture broth was obtained under sterile conditions from the BD BACTEC bottles and transferred into a sterile 1.5 ml tube (Eppendorf, Hamburg, Germany). These residual materials were stored at −80°C until further assessment in Germany. Traditional cultural growth on agar and biochemical differentiation of grown pathogens was performed in Kumasi as described below.

### Characterization of culture isolates

Bacteria from all positive blood culture bottles were grown on Columbia agar enriched with 5% sheep blood, MacConkey agar, and chocolate agar (Oxoid, Wesel, Germany) under aerobic conditions. Species identity was biochemically determined by preliminary microbiological procedures such as Gram staining, cytochrome oxidase testing, pyrase testing, clumping-factor latex agglutination, plasma coagulase testing, bacitracin-inhibition testing, and optochin testing, and by specific testing including latex agglutination for streptococci, *H*. *influenzae* type B, and *S*. *enterica*, as well as API biochemical detection systems 20E, 20NE, NH, and strep (bioMeriéux, Nürtingen, Germany) at the Kumasi Centre for Collaborative Research, Kumasi, Ghana. Gram-positive staphylococci and rod-shaped bacteria with colony morphology suggesting *Bacillus* spp., coagulase-negative staphylococci (clumping-factor negative), *Corynebacterium* spp., or *Micrococcus* spp. were considered as contaminants due to blood sample acquisition and were discarded without further discrimination. Repeated blood cultures that might have indicated potential etiological relevance of such isolates [23] were not obtained for organizational reasons.

### Sample preparation of analyzed specimens prior to PCR

The above-described residual materials from the automatically incubated blood culture materials were stored at −80°C prior to further preparation. As already detailed [8, 24], a cheap, simple, but laborious and time-consuming in-house centrifugation scheme, consisting of several steps, had been used to remove the erythrocytes and to enrich the target organisms. In brief, sedimentation of erythrocytes of 1 ml blood culture medium was performed at 140 × g for 10 minutes. The supernatant (800 μl) was transferred into another tube and the sediment was discarded. The 800 μl supernatant was mixed with 400 μl double-distilled water to lyse remaining erythrocytes. Bacteria were sedimented at 750 × g for 10 minutes and the supernatant was discarded. The pellet was re-suspended, washed in 1 ml double-distilled water, and again centrifuged at 750 × g for 10 minutes with the supernatant being discarded and the pellet being re-suspended in 200 μl double-distilled water. After incubation for 10 minutes at 95°C, 2 μl aliquots and 2.5 μl aliquots for subsequent PCR analysis were prepared. As previously demonstrated [8], this procedure was associated with a low sample inhibition rate of 4%. Prior to PCR, all samples that had undergone this enrichment of bacterial DNA were stored at −20°C.

### PCR

**Fig 1:**
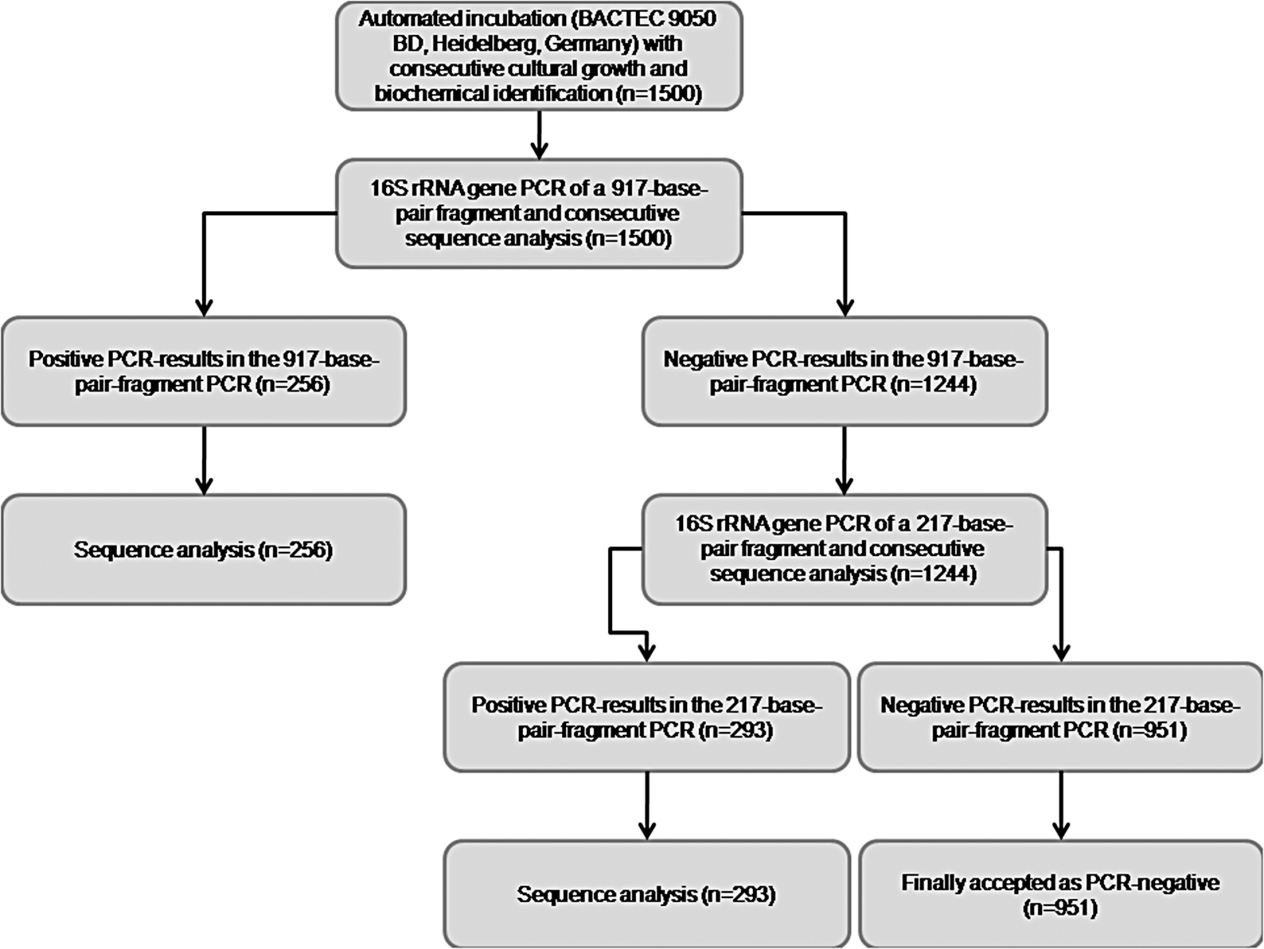
Flow chart visualizing the hierarchical sequence of PCR analyses performed. From all sequence results indicating *Streptococcus* spp. other than *Streptococcus agalactiae* (Lancefield group B) and *Streptococcus pyogenes* (Lancefield group A) in culture-negative, PCR-positive samples (n = 10): *sodA* gene PCR for the identification of *S*. *pneumonia*.

Two different, previously described SybrGreen 16S rRNA gene real-time PCRs were performed from the purified samples on a Rotor Gene 6000 real-time rotary analyzer (Corbett Life Science, Qiagen, Hamburg, Germany). First, an intermediate-fragment PCR protocol targeting a 917-base-pair fragment [25, 26] was performed (Fig 1, Table 1). Subsequently, a short-fragment PCR protocol targeting a 357-base-pair fragment as previously described for joint fluids [27] with minor modifications was added for all samples that remained negative with the first PCR (Fig 1, Table 1). The primers of this SybrGreen PCR were selected from a variety of tested oligonucleotides [27]. Positive (DNA of a clinical *Salmonella enterica* Typhimurium isolate in three decadic logarithmic dilution steps, the highest concentration adjusted to a cycle threshold [Ct]-value of 20) and negative (PCR-grade water) amplification controls were included in each run. If a positive signal was found in the negative control sample or vice versa, the whole PCR run was repeated.

**Table 1:** PCR protocols used for this study.

	917 bp 16S rRNA gene PCR	357 bp 16S rRNA gene PCR	*sodA* PCR
Master-mix			
Basic mix	2 μl LC Fast Start DNA Master SYBR Green 1 (10×) (Roche)	2.5 μl LC Fast Start DNA Master SYBR Green 1 (10x) (Roche)	10 μl HotStarTaq Mastermix (2x) (Qiagen)
MgCl	1.6 μl 25 mmol/l MgCl	2.0 μl 25 mmol/l MgCl	1.2 μl 25 mmol/l MgCl
Final MgCl concentration	3.0 mmol/l MgCl	3.0 mmol/l MgCl	3 mmol/l MgCl
Sample	2 μl	2.5 μl	2 μl
Forward primer	1 μl 16S8_27 5′-AGA-GTT-TGA-TCM-TGG-CTC-AG-3′ (8 pmol/μl)	0.5 μl 16s27F 5′-AAG-AGT-TTG-ATC-CTG-GCT-CAG-3′ (10 pmol/μl)	0.2 μl *sodA*-Up 5′-TRC-AYC-ATG-AYA-ARC-ACC-AT-3′ (10 pmol/μl)
Reverse primer	1 μl 16s907 5′-CCG-TCA-ATT-CMT-TTR-AGT-TT-3′ (8 pmol/μl)	0.5 μl 16s244 5′-CCC-ACT-GCT-GCC-TCC-CGT-AG-3′ (10 pmol/μl)	0.2 μl *sodA*-Down 5′-ARR-TAR-TAM-GCR-TGY-TCC-CAR-ACR-TC-3′ (20 pmol/μl)
Final concentration of each primer	0.4 pmol/μl	0.2 pmol/μl	*sodA*-Up: 0.1 pmol/μl, *sodA*-Down: 0.2 pmol/μl
PCR grade water	12.4 μl	17 μl	6.4 μl
Final volume	20 μl	25 μl	20 μl
Cycling			
Initial denaturation:	900 s 94°C	900 s 94°C	600 s 94C
Number of cycles	40×	40x	35x
Denaturation	20 s 94°C	20 s 94°C	20 s 94°C
Annealing	20 s 50°C	20 s 56°C	60 s 50°C
Amplification	60 s 72°C	45 s 72°C	60 s 72°C
Final step (one cycle)	300 s 72°C	300 s 72°C	30 s 94°C
	120 s 95°C	120 s 95°C	60 s 50°C
	30 s 70°C	30 s 75°C	600 s 72°C
Melting			
Ramp from	70°C–99°C	75°C–94°C	-
Temperature increase per step	0.6°C	0.6°C	-
Time of pre-melt conditioning on first step	90 s	90 s	-
Time for each step afterward	5 s	5 s	-

No inhibition control PCRs were performed. The previously determined inhibition rate of 4% [8] was adopted for this retrospective analysis as a rough assumption, although the 357-base-pair SybrGreen approach has been shown to be particularly prone to inhibition [27]. The cut-off for specific amplification was set at a Ct-value of 30.

As previously detailed [27], SybrGreen-based 16S rRNA gene PCR does not result in flat curves even with negative samples or negative control samples. Negative samples show a Ct-value around 30, so the cut-off was set at this value. Of note, this cut-off may vary for different PCR reagents and platforms. Using the SYBR Green PCR Master Mix reagent (Applied Biosystems, Waltham, Massachusetts, USA) on an ABI PRISM 7000 Seq detector system thermocycler (Applied Biosystems) for the 357-base-pair PCR, Ct-values of 38–39 were observed for negative control samples and Ct-values of 30–36 for positive samples [27]. On the Rotor Gene 6000 thermocycler, in contrast, the mean Ct-value even of presumed technical contaminations in culture-negative, PCR-positive samples was as low as 23.5, although low copy numbers are likely in case of such contaminations (S1 Table). For discrimination of positive and negative samples, melting-curve analysis is necessary. Positive samples show clear-cut peaks at 89°C (± 1.5°C standard deviation) for the 917-base-pair fragment PCR and 89.5°C (± 1°C standard deviation) for the 357-base-pair fragment PCR. The differences of the melting temperatures of DNA amplicons from different species are too low to allow for any discrimination as described [27]. Negative samples are associated with melting peaks at 78.5°C (± 1°C standard deviation) for the 917-base-pair fragment PCR and 83°C (± 0.5°C standard deviation) for the 357-base-pair fragment PCR or, in rare instances, no clearly distinguishable melting peak at all (S1 Fig). In such instances of irregular or missing melting peaks, no amplicon-specific bands are detectable in electrophoresis and sequencing attempts fail to provide bacterial sequences as described [27].

All samples containing *Streptococcus pneumoniae* DNA without *S*. *pneumoniae* in agar culture and all samples containing *Streptococcus* spp. DNA other than *S*. *pyogenes* or *S*. *agalactiae* were additionally analyzed by superoxide dismutase (*sodA*) gene sequencing as described [28] (Fig 1, Table 1) to ensure sufficient discrimination at species level within the *S*. *mitis* group.

### Sequencing and sequence analysis

Prior to sequencing, amplified DNA was purified using the NAT Clean-up/Nucleospin Extrakt II kit (Macherey & Nagel, Düren, Germany) according to the manufacturer’s instructions. Sequencing of the forward and reverse strands was performed by SeqLab Sequence Laboratories Göttingen GmbH (Göttingen, Germany). Sequence results were visually controlled, manually edited using the software “FinchTV” version 1.4.0 (Geospiza, Inc., Seattle, WA, USA) and “Nucleic acid sequence massager” (Attotron Biotechnologies Corporation, http://www.attotron.com/), and manually aligned. The resulting sequence was subsequently compared with deposited sequence information using the BLAST algorithm (http://blast.ncbi.nlm.nih.gov/Blast.cgi/).

Sequence analysis was performed as follows, taking account of the suggestions of the CLSI (Clinical and Laboratory Standards Institute) guideline MM18-A “Interpretive Criteria for Identification of Bacteria and Fungi by DNA Target Sequencing; Approved Guideline” [29]. In detail, ≥99% identity was demanded for identifications at species level, ≥97% identity for identifications at genus level. Worse scores due to poor sequence quality were considered as non-interpretable results. Also, 0.8% separation between different species was demanded.

No sequence information from this study was deposited in databases such as NCBI GenBank since no well-characterized strains were sequenced whose sequences might be a contribution to well-defined databases. Rather, sequence analysis was merely used for identification by sequence comparison with deposited sequence information.

### Interpretation in case of culture-negative, PCR-positive samples

In the case of positive PCR results in culture-negative samples, the possibility of technical contamination with bacterial DNA during sample preparation, including transfer from the blood culture bottles to the sample tubes and the DNA preparation itself, had to be considered. Technical contamination was distinguished from clinical contamination, i.e., the inoculation of vital bacteria from the skin into the blood culture bottle during sample acquisition, as described below. For the technical evaluation described here, only technical contamination was of interest.

The criteria for defining a positive PCR result without cultural growth as a technical contamination were unlikely etiological relevance, i.e., the identification of environmental species, and frequent detection of species by PCR only although cultural growth under aerobic conditions would have been expected for the respective species. There was necessarily a certain degree of subjectivity, as is typical for medical decisions. *Alicycliphilus* spp., Alphaproteobacteriaceae, *Bacillus halodurans*, *Bacterium* spp., *Balneomonas* spp., *Bradyrhizobium* spp., *Caulobacter* spp., *Cloacibacterium normanense*, cocci (not further defined), *Diaphorobacter* spp., Flexibacteriaceae, *Janibacter* spp., *Luteimonas* spp., *Riemerella* spp., *Sporichtya* spp., and *Thermonema* spp. as well as multiply superimposing sequences were assigned as technical contaminations. For the assignment of a sequence to the technical contamination group, sequence results of worse than 97% sequence identity with deposited reference sequences were accepted. Poor sequence quality in these cases was attributed to the low initial quantities of DNA that are typical for technical contaminations.

In addition to the group of suspected technical contaminations, there were several results of uncertain clinical importance. If the sequence quality of potentially etiologically relevant bacteria fell below the threshold of 97% sequence identity, the result was considered non-interpretable and thus uncertain. This group of uncertain results comprised further potential clinical contaminants, presumably resulting from non-sterile blood sample acquisition, and causative agents of transient bacteremia, e.g., due to gingival lesions of patients’ oral cavities, irrespective of the sequence quality. *Bacillus* spp., *Micrococcus* spp., *Mycobacterium* spp., *Staphylococcus epidermidis*, *Staphylococcus* spp., and *Streptococcus gallolyticus* ssp. *pasteurianus* were assigned to this group. Only one blood culture bottle per patient was available, so repeated detections could not be used for the assignment of potential etiological relevance.

Finally, a group of sequence results with potential etiological relevance in culture-negative samples was defined, which comprised sequences of etiologically relevant pathogens with a sequence quality ≥97%, thus at least allowing for an assignment at genus level and suggesting a need for a therapeutic intervention.

### Ct-value analysis

The 16S rRNA gene PCRs were not designed for quantification of DNA copies. Cycle threshold (Ct-)value analysis should allow a relative comparison of bacterial density in the samples. The Ct-value analysis was attempted to assess the likelihood of contamination, as these values are inversely correlated with DNA copy numbers in real-time PCR. Low copy numbers might indicate technical sample contamination.

### Statistics

The Ct-values of culture-positive and culture-negative samples with positive PCR results, and, within the latter, of the “technical contamination” subgroup and the “etiologically relevant” subgroup, were compared using the non-parametric Mann-Whitney test (GraphPad InStat, version 3.06, GraphPad Software Inc., La Jolla, CA, USA), because the test values did not pass normality testing (KS-test, GraphPad InStat). Two-tailed P-values were provided. These analyses were purely explorative and P-values are only descriptive, without defined cut-off values.

### Ethics

The study complied with the principles of the Helsinki Declaration of 1975 and with all subsequent amendments by the World Medical Assembly. Written informed consent of all study participants was obtained. If minors/children were enrolled in the study, written informed consent of the next to kin, caretakers, or guardians was obtained. Ethical approval for the study was guaranteed by the Committee on Human Research, Publications and Ethics, School of Medical Sciences, Kwame Nkrumah University, Kumasi, Ghana and the Institutional Review Board of the International Vaccine Institute, Seoul, Korea.

## Results

### Cultural growth

Bacterial growth was detected in 191 of 1500 aerobic blood cultures (12.7%). Strains of 93 culture-positive samples (6.2%) were considered as skin or environmental contaminants, including *Bacillus* spp., coagulase-negative staphylococci, *Corynebacterium* spp., *Micrococcus* spp., and *Rhizobium radiobacter*. The other 98 samples (6.5%) included potentially etiologically relevant non-fermenting, Gram-negative rod-shaped bacteria such as *Acinetobacter* spp., *Pseudomonas* spp., *Sphingomonas paucimobilis*; Enterobacteriaceae such as *Escherichia* spp., *Enterobacter* spp., *Klebsiella pneumoniae*, *Salmonella* spp., and *Serratia* spp.; Gram-positive cocci such as *Staphylococcus aureus* and *Streptococcus* spp.; and fastidious Gram-negative rod-shaped bacteria such as *Haemophilus influenzae* (Tables 2–4).

### Comparison of cultural growth and 16S rRNA gene PCRs

The combined use of both the 917 bp and 357 bp 16S rRNA gene PCRs in the stepwise diagnostic approach led to positive PCR results in all but two positive blood cultures. Moreover, PCR was positive in 360 blood culture samples without cultural growth (Table 2). The distribution of the detection of potentially etiologically relevant pathogens and presumed contaminants is detailed below as well as the identification pattern by the two-step PCR protocol.

**Table 2:** Summarized results of cultural growth and 16S rRNA gene PCR.

	917 bp 16S rRNA gene PCR			917 bp and 357 bp 16S rRNA gene PCR		
	Positive	Negative	Total	Positive	Negative	Total
Culture positive	157	34	191	189	2	191
Culture negative	99	1210	1309	360	949	1309
Total	256	1244	1500	549	951	1500

**Table 3:** Results of 917 bp 16S rRNA gene sequencing of culture-positive samples.

Results of culture		Results of the 917 bp 16S rRNA gene sequencing				
		Confirmed on				
Species	N	Species level (n/%)	Genus level only (n/%)	Negative (n/%)	Non-interpretable (n/%)	Different species (n/%)
Potentially relevant causes of sepsis						
*Acinetobacter* spp.	3	2/66.7%				1^d^/33.3%
Enterobacteriaceae^a^	57	28/49.1%	21/36.8%	2/3.5%		6^e^/10.5%
*H*. *influenzae*	1				1/100%	
*Pseudomonas* spp.	4			1/25%	1/25%	2^f^/50%
*S*. *agalactiae*	2		2 /100%			
*S*. *aureus*	9	1/11.1%	5/55.6%	3/33.3%		
*S*. *pneumoniae*	12	11/91.7%	1/8.3%			
*S*. *pyogenes*	1			1/100%		
Others^b^	9		1/11.1%	2/22.2%	2/22.2%	4^g^/44.4%
**Total**	**98**	**42/42.9%**	**30/30.6%**	**9/9.2%**	**4/4.1%**	**13/13.3%**
Potential contaminations during blood sample acquisition						
*Bacillus* spp.	10	3/30%	4/40%	3/30%		
Coagulase-negative staphylococci	65	17/26.2%	23/35.4%	12/18.5%	8/12.3%	5^h^/7.7%
*Corynebacterium* spp.	11		1/9.1%	5/45.5%	2/18.2%	3^i^/27.3%
Others^c^	7		1/14.3%	5/71.4%	1/14.3%	
**Total**	**93**	**20/21.5%**	**29/31.2%**	**25/26.9%**	**11/11.8%**	**8/8.6%**
**Combined total**	**191**	**62/32.5%**	**59/30.9%**	**34/17.8%**	**15/7.9%**	**21/11.0%**

^a^Comprising *Escherichia coli* (17), *Escherichia hermannii* (1), *Enterobacter cloacae* (1), *Klebsiella pneumoniae* (5), *Salmonella enterica* (11), *Salmonella* spp. (8), *Serratia* sp. (1) according to biochemical identification.

^b^Including Gram-negative, not further identified coliform bacteria (3), Gram-negative, not further identified non-fermentative rod-shaped bacteria (1), *Sphingomonas paucimobilis* (2), *Streptococcus* Lancefield Group G (2), viridians group streptococci (1)

^c^Including *Micrococcus* spp. (6), and *Rhizobium radiobacter* (1).

^d^ *Bacillus* sp. identified by PCR and sequencing.

^e^Biochemically confirmed *Escherichia coli* not differentiable between *Escherichia coli* and *Shigella* spp. (4), biochemical result *Serratia* sp. vs. sequencing result *Enterobacter* sp. (1), biochemical result *Klebsiella pneumoniae* vs. sequencing result *Enterobacter* sp. (1).

^f^Biochemically identification *Pseudomonas luteola* (2) vs. sequence-based identification as *Acinetobacter baumannii* (1) and *Acinetobacter* sp. (1), respectively.

^g^Cultural suspicion of Gram-negative, coliform bacteria (2) vs. sequence results *Bacillus* sp. (1) and *Microbacterium* sp. (1), biochemically identified *Sphingomonas paucimobilis* (2) vs. sequence results *Paenibacillus xylanilyticus* (1) and *Microbacterium oxydans* (1).

^h^Cultural suspicion of coagulase-negative staphylococci (5) vs. sequencing results *Bacterium* spp. (2), *Micrococcus luteus* (1), *Sphingomonas* sp. (1), and *Staphylococcus aureus* (1).

^i^Cultural suspicion of *Corynebacterium* spp. (3) vs. sequencing results *Arthrobacter* spp. (2) and *Microbacterium* sp. (1).

**Table 4:** Results of 917 bp 16S rRNA gene sequencing and consecutive 357 bp 16S rRNA gene sequencing in case of negative 917 bp 16S rRNA gene PCR results of culture-positive samples.

Results of culture		Results of 917 bp 16S rRNA gene sequencing and consecutive 357 bp 16S rRNA gene sequencing in case of negative 917 bp 16S rRNA gene PCR results				
		Confirmed on				
Species	N	Species level (n/%)	Genus level only (n/%)	Negative (n/%)	Non-interpretable (n/%)	Different species (n/%)
Potentially relevant causes of sepsis						
*Acinetobacter* spp.	3	2/66.7%				1^d^/33.3%
Enterobacteriaceae^a^	57	28/49.1%	21/36.8%			8^e^/14.0%
*H*. *influenzae*	1				1/100%	
*Pseudomonas* spp.	4				2/50%	2^f^/50%
*S*. *agalactiae*	2		2 /100%			
*S*. *aureus*	9	2/22.2%	5/55.6%		2/22.2%	
*S*. *pneumoniae*	12	11/91.7%	1/8.3%			
*S*. *pyogenes*	1	1/100%				
Others^b^	9		2/22.2%		2/22.2%	5^g^/55.6%
**Total**	**98**	**44/44.9%**	**31/31.6%**		**7/7.1%**	**16/16.3%**
Potential contaminations during blood sample acquisition						
*Bacillus* spp.	10	3/30%	5/50%	1/10%	1/10%	
Coagulase-negative staphylococci	65	18/27.7%	24/36.9%		10/15.4%	13^h^/20%
*Corynebacterium* spp.	11		1/9.1%		5/45.5%	5^i^/45.5%
Others^c^	7		1/14.3%	1/14.3%	2/28.6%	3^j^/42.9%
**Total**	**93**	**21/22.6%**	**31/33.3%**	**2/2.2%**	**18/19.4%**	**21/22.6%**
**Combined total**	**191**	**65/34.0%**	**62/32.5%**	**2/1.0%**	**25/13.1%**	**37/19.4%**

^a^Comprising *Escherichia coli* (17), *Escherichia hermannii* (1), *Enterobacter cloacae* (1), *Klebsiella pneumoniae* (5), *Salmonella enterica* (11), *Salmonella* spp. (8), *Serratia* sp. (1) according to biochemical identification.

^b^Including Gram-negative, not further identified coliform bacteria (3), Gram-negative, not further identified non-fermentative rod-shaped bacteria (1), *Sphingomonas paucimobilis* (2), *Streptococcus* Lancefield Group G (2), viridians group streptococci (1).

^c^Including *Micrococcus* sp. (6), and *Rhizobium radiobacter* (1).

^d^ *Bacillus* sp. identified by PCR and sequencing.

^e^Biochemically confirmed *Escherichia coli* not differentiable between *Escherichia coli* and *Shigella* sp. (4) based on 16S rRNA gene sequencing or identified as *Enterobacter* sp. (1), biochemical result *Serratia* sp. vs. sequencing result *Enterobacter* sp. (1), biochemical result *Klebsiella pneumoniae* vs. sequencing result *Enterobacter* sp. (1), biochemical result *Salmonella* sp. vs. sequencing result *Diaphorobacter* sp. (1).

^f^Biochemically identification *Pseudomonas luteola* (2) vs. sequence-based identification as *Acinetobacter baumannii* (1) and *Acinetobacter* sp. (1), respectively.

^g^Cultural suspicion of Gram-negative, coliform bacteria (2) vs. sequence results *Bacillus* sp. (1) and *Microbacterium* sp. (1), cultural suspicion of Gram-negative, non-fermentative rod-shaped bacteria (1) vs. sequence result *Nesterenkonia* sp., biochemically identified *Sphingomonas paucimobilis* (2) vs. sequence results *Paenibacillus xylanilyticus* (1) and *Microbacterium oxydans* (1).

^h^Cultural suspicion of coagulase-negative staphylococci (13) vs. sequencing results *Bacterium* spp. (2), *Corynebacterium* sp. (1), *Diaphorobacter* spp. (3), *Haematobacter massiliensis* (1), *Kocuria* sp. (1), *Micrococcus luteus* (1), *Micrococcus* sp. (1), *Pseudomonas* sp. (1), *Sphingomonas* sp. (1), and *Staphylococcus aureus* (1).

^i^Cultural suspicion of *Corynebacterium* spp. (5) vs. sequencing results *Arthrobacter* spp. (2), *Diaphorobacter* spp. (2), *Massilia aurea* (1), and *Microbacterium* sp. (1).

^j^Cultural suspicion of *Micrococcus* spp. (3) vs. sequence results *Diaphorobacter* sp. (1), *Kocuria marina* (1), and *Staphylococcus* sp. (1).

### Concordance of 16S rRNA gene sequencing results and cultural growth

From all 191 isolated strains, the 917 bp fragment 16S rRNA gene PCR with consecutive sequencing from blood culture broth led to concordant results for 62 strains (32.5%) at species level and a further 59 strains (30.9%) at genus level (Table 3). In 21 instances (11.0%), culture and PCR/sequencing led to discordant results. In 15 cases (7.9%), sequence quality was too poor for an assignment at species or genus level. In 34 instances (17.8%), comprising 4 Gram-negative, rod-shaped bacteria (2.1%), PCR remained negative (Table 3). The 917 bp fragment 16S rRNA gene PCR was positive in 99 additional samples that remained negative in BACTEC automated blood culture (Table 5).

**Table 5:** Results of the 917 bp 16S rRNA gene PCR and the 357 bp 16S rRNA gene PCR from culture-negative samples.

Species	Positive by 917 bp 16S rRNA gene PCR	Negative by 917 bp 16S rRNA gene PCR but positive by 357 bp 16S rRNA gene PCR
Potentially relevant causes of sepsis		
*Acinetobacter* spp.	5	
*Brevundimonas* spp.	1	
*Campylobacter* spp.	1	
*Clostridium* spp.		1
Enterobacteriaceae^a^	29	12
*Moraxella* spp.		1
*Neisseria* spp.		1
*Pseudomonas* spp.	1	1
*Stenotrophomonas* spp.	1	4
*Streptococcus pneumoniae*	1^d^	2^e^
*Streptococcus pyogenes*	1	
**Total**	**40**	**22**
Identifications of uncertain clinical importance and contaminations with environmental samples		
Identifications of uncertain clinical relevance^b^	17	33
Probable contaminations during sample preparation (technical contaminations)^c^	42	206
**Total**	**59**	**239**
**Combined total**	**99**	**261**

^a^Comprising *Escherichia coli* (4), *E*. *coli*/*Shigella* spp. (2), *Escherichia* sp. (1), *Enterobacter* spp. (9), *Ewingella americana* (2), *Ewingella* spp. (3), *Klebsiella pneumoniae* (1), *Salmonella enterica* (9), *Salmonella* spp. (2), *Serratia* spp. (5), *Yersinia* spp. (3).

^b^Comprising potential contaminations during sample acquisition, bacteria which might be associated with transient bacteremia, and sequences with too poor quality for identifications even on genus level, i.e., *Bacillus* sp. (1), *Micrococcus* sp. (1), *Mycobacterium* sp. (1), *Staphylococcus epidermidis* (2), *Staphylococcus* spp. (4), *Streptococcus gallolyticus* ssp. *pasteurianus* (1), non-interpretable results due to poor sequence quality (40).

^c^Comprising *Alicycliphilus* sp. (1), Alphaproteobacterium (4), *Bacillus halodurans* (1), *Bacterium* sp. (1), *Balneomonas* sp. (1), *Bradyrhizobium* spp. (5), *Caulobacter* spp. (26), *Cloacibacterium normanense* (2), cocci (not further defined) (1), *Diaphorobacter* spp. (155), Flexibacteriaceae (1), *Janibacter* sp. (1), *Luteimonas* sp. (1), multiply superimposing sequences (45), *Riemerella* sp. (1), *Sporichtya* sp. (1), *Thermonema* sp. (1).

^d^In addition to this one detection of *S*. *pneumoniae* DNA, *sodA* sequencing identified *S*. *pneumoniae* DNA in one additional culture-negative sample, in which 16S rRNA gene sequencing did not allow for an identification even on genus level due to poor sequence quality.

^e^Confirmed by *sodA* sequencing.

The 357 bp fragment 16S rRNA gene PCR that was performed with all samples that remained negative in the 917 bp fragment 16S rRNA gene was positive in an additional 293 instances (Fig 1). It increased the number of concordant identifications at species level by 3 of 34 (8.8%) to a total of 65 (34.0%), the number of concordant identifications at genus level by 3 of 34 (8.8%) to a total of 62 (32.5%), the number of discordant results by 16 of 34 (47.1%) to a total of 37 (19.4%), and the number of non-interpretable sequencing results by 10 of 34 (29.4%) to a total of 25 (13.1%). Only 2 samples (1.0%) with cultural growth of *Bacillus* spp. and *Micrococcus* spp., respectively, remained negative in both PCRs (Tables 2 and 4). The number of additional positive PCRs in culture-negative samples was increased by 261 to a total of 360 (Table 5).

Among the isolates with suspected clinical relevance, the 917 bp fragment 16S rRNA gene PCR failed to identify 1 *E*. *coli*, 1 *P*. *aeruginosa*, 1 *Pseudomonas* sp., 1 *Salmonella* sp., 8 *S*. *aureus* at species level, 1 *S*. *pyogenes*, 2 *Streptococcus* spp. Lancefield type G, and 1 *Streptococcus* sp. of the *viridians* group, i.e., 16 out of 98 strains (16.3%), even if identification of 2 *S*. *agalactiae* and 1 *S*. *pneumoniae* at genus level only is accepted as sufficient for a therapeutic decision (Table 3). Among these failed identifications, the 357 bp fragment 16S rRNA gene PCR additionally recovered 1 *S*. *aureus* and 1 *S*. *pyogenes* at species level, and 1 *Streptococcus* sp. Lancefield type G at genus level (Table 4), decreasing the number of missed important strains to 13 out of 98 (13.3%).

In three samples, from which the culture isolates were identified as coagulase-negative *Staphylococcus* spp., sequencing resulted in the identification of *Pseudomonas* sp., *S*. *aureus*, and *Sphingomonas* sp., respectively. Only one 1 out of 11 supposed *Corynebacterium* spp. isolates was confirmed as such on sequence basis, while in 2 of these samples *Arthrobacter* spp., potential causes of infectious endocarditis [30], were identified by sequence analysis. In one sample with *Acinetobacter* sp. in culture, sequencing detected *Bacillus* sp., a likely confusion as *Acinetobacter* spp. are pleomorphic Gram-labile rod-shaped bacteria. In 5 instances of etiologically relevant *S*. *aureus* growth, 2 instances of *S*. *agalactiae* growth and 1 instance of *S*. *pneumoniae* growth, sequencing allowed identification at genus level only (Tables 3 and 4).

### Additional positive sequence results in culture-negative samples

Regarding the additional sequence results of PCR-positive but culture-negative samples, the 917 bp fragment 16S rRNA gene PCR showed 40 out of 99 positive results (40.4%) with a suspected therapeutic relevance; the 357 bp fragment 16S rRNA gene PCR showed a further 22 out of 261 results (8.4%). Sequences of non-fermentative Gram-negative rod-shaped bacteria such as *Acinetobacter* spp., *Brevundimonas* spp., *Pseudomonas* spp., *Stenotrophomonas* spp.; Enterobacteriaceae such as *Escherichia* spp., *Enterobacter* spp., *Ewingella* spp., *Klebsiella pneumoniae*, *Salmonella* spp., *Serratia* spp., and *Yersinia* spp.; Gram-positive *S*. *pneumoniae* and *S*. *pyogenes*; systemically spreading Gram-negative cocci, i.e., *Neisseria* spp. and *Moraxella* spp., and micro-aerophilic pathogens such as *Campylobacter* spp. or anaerobic pathogens like *Clostridium* spp. were subjectively considered as etiologically relevant (Table 5).

Additional information regarding the presumptive etiological agent of sepsis was obtained for 2.7% (40/1500) of the samples by the 917 bp fragment 16S rRNA gene PCR and by the combined use of both PCRs for as many as 4.1% (62/1500), thus increasing the proportion of suspected etiologically relevant blood culture pathogens detected by the combination of culture and PCR to a total of 10.7% of all 1500 analyzed samples. Samples with suspected technical contaminations in 16S rRNA gene PCR were 2.8% (42/1500) in the 917 bp fragment 16S rRNA gene PCR and 16.5% (248/1500) with the combined use of both PCRs (Table 5).

### Ct-value analysis

Comparison of the Ct-values of positive PCRs of the groups with culture-positive samples and culture-negative samples showed significantly lower Ct-values for the culture-positive group in the Mann-Whitney test for both PCR assays (S1 Table). However, the mean values were so close to each other that no cut-off value could be defined. Within the PCR-positive, culture-negative group, the subgroups with suspected etiologically relevant pathogens and with presumed technical contaminants showed different reaction patterns for the two PCR assays. The group with potentially etiologically relevant pathogens showed significantly lower Ct-values than that with suspected technical contaminants for the 917 bp 16S rRNA gene PCR. For the 357 bp 16S rRNA gene PCR, the result was exactly the opposite and also significant (S1 Table), not allowing for any further interpretation.

### *sodA* sequencing

*sodA*-sequencing confirmed 3 *S*. *pneumoniae* that were identified by 16S rRNA gene PCR in culture-negative samples and identified *S*. *pneumoniae* DNA in a further culture-negative sample in which the quality of the 16S rRNA sequence was too poor for an identification even at genus level (Table 5).

## Discussion

In the study described here, an intermediate-fragment real-time 917 bp 16S rRNA gene PCR protocol [25, 26] and also a short-fragment 357 bp 16S rRNA gene PCR protocol [27] were applied to a subset of blood culture materials from a study conducted in Ghana [4, 8, 21, 22]. From the optimization efforts mentioned including testing of various primer pairs to achieve optimum results from articular fluids of septic arthritis patients [27], the superiority of the 357 bp 16S rRNA gene PCR in terms of sensitivity from primary patient materials was assumed. In contrast, the 917 bp approached is routinely used for sequencing from culture material [25, 26] in our laboratory and was not especially designed for PCR from primary patient materials. Accordingly, the 357 bp PCR was used in a stepwise approach if the 917 bp PCR, allowing better discrimination due to the longer sequence fragment, remained negative. No additional direct comparison of the two protocols was either intended or possible, because the two sets of primers were not used on the same specimens. If the 357-base pair primers had been tried first, it is likely that the 917-base pair ones tried second would have picked up some positives that the 357-base pair primers missed.

The analysis was undertaken to determine the concordance between the diagnosis after classical blood culture and that after PCR of automatically incubated blood and sequencing. The hypothesis was that culture might miss a high proportion of easily killed blood culture pathogens, for example, due to suboptimal storage and transport conditions in the tropics as previously discussed [8]. Under these circumstances, multiplex real-time PCR for enteropathogenic bacteria resulted in an additional one-third of *Salmonella* spp. detections in materials from this study [8]. By our analysis we aimed to determine whether 16S rRNA gene sequencing might result in better sensitivity for epidemiological studies in the tropics in times of decreasing prices for Sanger sequencing, and, in the intermediate term, might even be a diagnostic option. When interpreting the results, one has to bear in mind that neither the presence of vital bacteria nor positive 16S rRNA gene PCR indicates clinical relevance, because both are prone to contamination and the latter to amplification of non-vital bacterial DNA fragments.

Sequencing from blood culture broth allowed for the identification of one-third of the isolated bacteria at species level and another one-third at genus level. In most cases, sequence-based identification at genus level would have led to the same therapeutic decisions concerning antibiotic therapy as identification at species level, if local resistance surveillance information was available. Of course, sequence-based identification is no replacement for resistance testing.

Genus-level identifications are usually acceptable for estimation of the etiological relevance of most Enterobacteriaceae. However, the inadequate identification of 5 *S*. *aureus* isolates only as *Staphylococcus* spp. is troubling, because *Staphylococcus* spp. are common contaminants from the cutaneous flora [23]. Accordingly, a correct sequence-based identification of only 2 out of 9 biochemically confirmed *S*. *aureus* at species level is an unacceptably poor result. In contrast, only 1 *S*. *aureus* detection by PCR was missed by agar culture. Similarly, the incomplete identification of 2 *S*. *agalactiae* strains and 1 *S*. *pneumoniae* strain as *Streptococcus* spp. is unacceptable, as both species can be associated with meningitis, particularly in children.

The 37 occasions showing a different species diagnosis in blood culture and on molecular analysis were difficult to interpret. Probable contaminants such as *Bacillus* spp., coagulase-negative staphylococci, *Corynebacterium* spp., or *Micrococcus* spp. were only crudely assessed after positive blood culture, i.e., by colony morphology and Gram staining. Biochemical classification was usually not done. It is likely, for example, that an atypical-looking *S*. *aureus* has been confused with a coagulase-negative staphylococcus, or an *Arthrobacter* sp. with *Corynebacterium* sp. The confusion of potentially etiologically relevant species such as *Arthrobacter* spp. [30] with skin contaminants such as *Corynebacterium* spp. may be deleterious for the patient. Discordant results regarding Gram-negative rod-shaped bacteria might reflect occasional chance errors in interpreting API results or, alternatively, atypical reaction patterns of individual isolates in the tropics. However, the infrequently observed discordances between biochemical identification and sequencing within the Enterobacteriaceae and non-fermentative rod-shaped bacteria would usually have been of minor therapeutic relevance. Gram-lability (e.g., due to antibiotic therapy) might have resulted in the occasionally observed confusion of Gram-positive and Gram-negative organisms.

Altogether, sequencing results discordant with culture by the 917 bp PCR seemed more likely to identify etiologically relevant pathogens than discordant results of the 357 bp PCR. For the latter, the primers seem to have bound to technical contaminants such as *Diaphorobacter* spp. more readily than to the genuine pathogen. This problem of selective binding affinities of pan-eubacterial primers is known from microbiome projects [31].

The noticeably low Ct-values of samples with suspected technical contaminations, Ct-values of 21–23, suggesting relatively high loads of contaminant DNA, remain puzzling. Interestingly, similarly low Ct-values due to amplification of contaminant DNA have recently been observed for panfungal PCRs from paraffin-embedded, formalin-fixed tissue of patients with invasive tropical mycoses (see supplementary materials of [32]). Both the source and the extent of any potential reproduction of contaminants prior to sample freezing remain unclear and this cannot be resolved by the data assessed, which is an undeniable limitation of the study. The plastic tubes where the samples had been deposited in Ghana or the tubes that were used for the centrifugation process might be potential sources, but this is merely speculative. Such a common source might explain the low heterogeneity of the observed technical contaminations, next to preferential primer binding affinities to certain contaminants as previously observed [31, 32]. The analysis of empty tubes might have confirmed or excluded this hypothesis, but was not performed. In the light of this, we recommend that negative controls from all charges of disposable materials that are used for the pre-analytic process should be included in similar future studies.

The proportion of non-interpretable sequences due to poor sequence quality or overlays of different sequences in contaminated samples or samples containing more than one pathogen was pleasingly low. Although software-based algorithms to separate different overlaying sequences have been described [33], we did not apply such procedures.

The negative PCR results observed in spite of cultural growth are troubling. While the negative 917 bp PCR results for Gram-negative bacteria were within the known and expected inhibition rate of 4% of the analyzed samples [8], 25% of Gram-positive bacteria were missed. A total of 83.4% of these 25% Gram-positive bacteria were presumably contaminations occurring during sample acquisition.

The centrifugation-based DNA preparation protocol that we used is cheap and easy to apply. However, it contains no steps to break the murein wall of Gram-positive bacteria, leading to low amounts of DNA of Gram-positive pathogens. Such centrifugation-based losses can explain the high percentage of missed Gram-positive bacteria. For the same reason, MALDI-TOF-MS (matrix-assisted laser-desorption-ionization time-of-flight mass spectrometry) from blood culture materials shows reduced diagnostic accuracy for Gram-positive pathogens [24]. Future studies should address this limitation by including appropriate digestion steps.

Although the centrifugation-associated loss of Gram-positive pathogens, which tend to form clusters with similar sedimentation characteristics as erythrocytes, had been expected based on previous study results [24], the cheap and easy-to-apply centrifugation approach that had led to acceptable results with Gram-negative pathogens [8] was chosen for the analysis. Alternative manual nucleic acid extraction with the Qiamp DNA Mini Kit (Qiagen, Hilden, Germany) and automated extraction using a QIAsymphony (Qiagen) system had been associated with considerable PCR inhibition if directly applied with blood culture material from BD blood culture bottles in previous tests before the study was conducted (data not shown). Therefore, commercial nucleic acid extraction kits were not considered any further for use in the study.

The 917 bp PCR identified the majority of pathogens in culture-positive samples, while additional etiologically relevant detections due to the subsequent use of the 357 bp PCR were almost negligible. Regarding culture-negative, PCR-positive samples, the proportion of presumably etiologically relevant detections was increased by one-third from 2.7% to 4.1% by the addition of the short-fragment 357 bp PCR. Compared with culturally grown bacteria with presumptive etiological relevance, there was an increase from 98/1500 (6.5%) to 160/1500 (10.7%) due to the combination of culture and 16S rRNA gene sequencing.

These data are well in line with our previous study showing an increased yield of *Salmonella* spp.-positive samples by one-third with a real-time multiplex PCR for enteropathogenic bacteria from the same materials, possibly reflecting suboptimal storage and transport conditions of the blood culture bottles in the tropics as discussed [8]. A disproportionately increased detection of particularly easily killed bacteria such as *S*. *pneumoniae* or *H*. *influenzae* [11–15] was not observed, however. 16S and *sodA* sequencing led to an increase of *S*. *pneumoniae* detections by one-third from 12 to 16, in the same range as for salmonellae. Additional *H*. *influenzae*-positive samples were not detected by sequencing, possibly reflecting a successful vaccination scheme in Ghana, where invasive *H*. *influenzae* infections were formerly more common [34]. Accordingly, conclusions cannot be drawn regarding the sensitivity of PCR for the purpose of *H*. *influenzae* detection in blood culture.

Assignment of etiological relevance in culture-negative samples is often debatable. For example, the detection of *Acinetobacter* spp. was considered potentially relevant because bacteremia due to *Acinetobacter* spp. in severely ill patients has been described [35], although *Acinetobacter* spp. are a common colonizer of the intact skin as well [36]. *Neisseria* sp. was classified as a pathogen on the assumption that it was *Neisseria meningitides* or *N*. *gonorrhoeae*, and *Moraxella* sp. was considered to be *Moraxella catarrhalis*, although contamination with oral flora in the course of the sample preparation cannot be excluded. In contrast, DNA remnants of alpha-hemolytic streptococci were assigned to uncertain clinical relevance as a potential consequence of inappropriate oral hygiene of the patient, although infectious endocarditis is not excluded in such instances. Similarly, the clinical impact of the not-further-defined *Mycobacterium* sp. was interpreted with care. Admittedly, the choice may have included a certain degree of subjectivity and might have been decided otherwise in a few instances, reflecting the difficulties in interpreting the clinical relevance of microbiological diagnostic results.

The fact that only one blood culture bottle per patient was inoculated is an undeniable limitation of the study because it does not allow for any estimation of potential etiological relevance if typical skin colonizers are detected. Accordingly, conclusions on the frequency of blood stream infections due to typical skin colonizers in Ghana cannot be drawn from this study and would go beyond the scope of this work.

The proportion of samples with presumed technical contamination increased from 2.8% in the 917 bp PCR to 16.5% with the addition of the short-fragment 357 bp PCR. This observation emphasizes the disadvantages of highly sensitive short-range PCRs in an environment of contamination risk [37], because the detection of technical contaminations may distract from the real cause of the patients’ pathology. Accordingly, the importance of a thorough interpretation of sequence results after pan-eubacterial PCR has to be stressed. As stated above, potential sources of contamination might be the transfer from the blood culture bottle to the storage tube in Ghana, or the centrifugation-based DNA enrichment in Germany. In ultrasensitive 16S rRNA gene sequence PCRs, even DNA contamination of PCR reagents may play a critical role in false-positive detections [38, 39]. Automated DNA extraction procedures that are applicable to blood culture broths may reduce contamination in future studies. Although a cheap centrifugation-based sample preparation may be sufficient for specific PCRs [8], pan-eubacterial PCRs seem to be too susceptible to contamination for such approaches. As discussed above, semiquantitative comparison of Ct-values was not a reliable marker for *a priori* discrimination of technical contaminations from vital pathogens in our analysis.

The high rate of observed technical contaminations limits the undeniable benefit of 62 out of 160 (38.8%) more detections of potentially etiologically relevant organisms. The interpretation of the results requires experience in medical microbiology and close cooperation with the clinician in charge. As an element of such cooperation, the clinician should provide any information that might affect the sensitivity of blood culture on the delivery note, e.g., about current antibiotic therapies and about any deviations from the manufacturer’s instructions during the inoculation of blood culture bottles. As is typical for microbiological routine diagnostics, such information was provided infrequently and was of poor quality during this assessment, representing a further limitation of the study. Accordingly, any corresponding analyses would be biased and therefore were not performed. However, the negligible Ct-value differences of culture-positive and culture-negative samples with positive PCR results suggest that lack of bacterial growth in the blood culture bottle was not a major reason for the observed discrepancy between PCR results and cultural results.

It remains speculative and cannot be assessed from the available data how many blood culture bottles actually suffer not from lack of growth but from exhausted growth. However, the low Ct-values obtained for culture-negative, PCR-positive samples suggest a considerable proportion. Indeed, the methods used for analyzing the blood culture bottles on the BACTEC instrument were sub-optimal. In fact, the bottles were put on the instrument regardless of whether they had already turned positive in the time between collection and the arrival in the laboratory. This is an undeniable limitation of the study and a highly likely reason why so many Enterobacteriaceae, which are easy to grow, difficult to kill, and usually signal positive in blood culture bottles monitored automatically after about 24 hours, were missed by culture. That these specimens were called negative for growth simply because there was no positive signal without consequent blind culturing on agar in all instances is an error in culture technique that should not be repeated in future sepsis studies in the tropics. This error can be corrected easily and cheaply by blind sub-culturing at arrival and at 24–48 hours incubation in the laboratory, a procedure that is within the constraints found even in resource-limited settings.

Additional procedural shortcomings have to be stressed that limit the interpretability of the results and might in part explain the comparably poor performance of the cultural approach. First of all, the lack of continuous control and monitoring of both storage and transport time and temperature may be an important reason for the discrepancy between culture and PCR/sequencing methods. Although missing data do not allow for a thorough assessment, it is highly likely that there is a correlation of discrepancies between culture and PCR/sequence methods in the specimens with the length of transport times. Further, primary Gram-staining should have been done from all positive blood culture bottles. This might have allowed for the isolation of difficult to grow *Campylobacter* spp., which were identified by PCR alone in the previous study [8], by creating micro-aerophilic growth conditions and using appropriate media such as Karmali agar (Oxoid), if any tiny curved Gram-negative structures were observed. Further, sub-culturing conditions of the positive blood culture bottles might have been improved by using a CO_2_ incubator to facilitate the growth of *Neisseria* spp., *Haemophilus* spp., and *S*. *pneumoniae*.

Although the continuous provision of supply for a CO_2_ incubator may be difficult in resource-limited tropical settings, monitoring of storage and transport times and conditions and primary Gram-staining of material from all positive blood culture bottles always seems possible with realistic effort. If adequate storage and transport conditions cannot be maintained for logistic reasons, so that exhausted growth within the blood culture bottle has to be expected, blind sub-culturing at arrival and at 24–48 hours incubation in the laboratory should always be performed in order not to miss vital pathogens. With implementation of these simple and cheap procedures, it should be possible to increase the performance of culture-based blood culture diagnostics in the tropical setting and to achieve better cultural results than in the study described here.

Promising alternative technologies are emerging. MALDI-TOF-MS shows reliable results when applied to automatically incubated blood culture samples [24], particularly after optimized prior centrifugation protocols [40]. However, the performance of MALDI-TOF-MS remains unclear in samples in which the blood culture pathogen dies relatively quickly after sample acquisition due to inappropriate transport and storage conditions, so DNA-based approaches might be superior. However, particularly in cases of low DNA amounts, contamination-free sample preparation is essential for DNA-based diagnostic assays.

Regarding the currently observed contamination rates, the interpretation of the results requires skills and expertise in clinical microbiology and infectious diseases. Nevertheless, an increase in potentially relevant diagnostic findings in blood culture materials by more than one-third justifies further attempts to optimize sequence-based diagnostic techniques to identify blood culture pathogens in septic patients, at least for epidemiological studies under unfavorable pre-analytic conditions. If pre-analytic conditions for cultural diagnostic approaches can be optimized, however, the additional benefit of the sequencing approach will presumably be less. For settings with restricted pre-analytic conditions, such as in resource-limited tropical areas, the results of this study and the previously published one [8] challenge the status of traditional blood culture based on automated incubation as the gold standard in sepsis diagnostics.

As well as contamination risks, an awkward procedure, relatively high costs of USD 30–50 per sample, and a time-to-result of several days limit the use of the method we describe for sepsis patients in resource-limited tropical settings. Even if the workflow is optimized, PCR and sequencing will demand 2–3 days for the long-fragment approach, 3–5 days if the short-fragment PCR/sequencing approach has subsequently to be added due to a negative result in the long-fragment PCR. For epidemiological assessments, for which time-to-result is less important, the price is still considerable. However, in times of progressive automation and falling sequencing costs, making even next-generation sequencing (NGS) runs already available for a few hundred US dollars nowadays [41], these problems might be overcome in the next few years.

## Supporting Information

S1 Fig Specific amplification (colored curves) in positive samples and non-specific signals in a negative sample (black curves). Amplification is depicted in the upper panel, melting curve analysis in the lower panel. The atypical melting peak in the negative sample (black curve) can be discriminated from the specific peaks of the positive samples (colored curves).
(TIF)

S1 Table Comparison of Ct-values in PCR-positive samples with and without cultural growth. (DOC)

## References

[1] Berkley JA, Lowe BS, Mwangi I, Williams T, Bauni E, Mwarumba S, et al (2005) Bacteremia among children admitted to a rural hospital in Kenya. N Engl J Med 352: 39–47. 15635111 10.1056/NEJMoa040275

[2] Reddy EA, Shaw AV, Crump JA (2010) Community-acquired bloodstream infections in Africa: a systemic review and meta-analysis. Lancet Infect Dis 10: 417–432. 10.1016/S1473-3099(10)70072-4 20510282 PMC3168734

[3] Deen J, von Seidlein L, Andersen F, Elle N, White NJ, Lubell Y (2012) Community-acquired bacterial bloodstream infections in developing countries in south and southeast Asia: a systematic review. Lancet Infect Dis 12: 480–487. 10.1016/S1473-3099(12)70028-2 22632186

[4] Nielsen MV, Sarpong N, Krumkamp R, Dekker D, Loag W, Amemasor S, et al (2012) Incidence and characteristics of bacteremia among children in rural Ghana. PLoS ONE 7: e44063 10.1371/journal.pone.0044063 22970162 PMC3438186

[5] Schaberg DR, Culver DH, Gaynes RP (1991) Major trends in the microbial etiology of nosocomial infection. Am J Med 91: 72–75. 10.1016/0002-9343(91)90346-y 1928195

[6] Geerdes-Fenge HF, Ziegler D, Lode H, Hund M, Loehr A, Fangmann W, et al (1992) Septicemia in 980 patients at a university hospital in Berlin: prospective studies during 4 selected years between 1979 and 1989. Clin Infect Dis 15: 991–1002. 1457672 10.1093/clind/15.6.991

[7] Wisplinghoff H, Bischoff T, Tallent SM, Seifert H, Wenzel RP, Edmond MB (2004) Nosocomial bloodstream infections in US hospitals: analysis of 24,179 cases from a prospective nation-wide surveillance study. Clin Infect Dis 39: 309–317. 15306996 10.1086/421946

[8] Frickmann H, Dekker D, Boahen K, Acquah S, Sarpong N, Adu-Sarkodie Y, et al (2013) Increased detection of invasive enteropathogenic bacteria in pre-incubated blood culture materials by real-time PCR in comparison with automated incubation in Sub-Saharan Africa. Scand J Infect Dis 45: 616–622. 10.3109/00365548.2013.777777 23547567

[9] Chaudhry R, Laxmi BV, Nisar N, Ray K, Kumar D (1997) Standardisation of polymerase chain reaction for the detection of *Salmonella* Typhi in typhoid fever. J Clin Pathol 50: 437–439. 9215131 10.1136/jcp.50.5.437 PMC499950

[10] Massi MN, Shirakawa T, Gotoh A, Bishnu A, Hatta M, Kawabata M (2005) Quantitative detection of *Salmonella enterica* serovar Typhi from blood of suspected typhoid fever patients by real-time PCR. Int J Med Microbiol; 295: 117–120. 15969472 10.1016/j.ijmm.2005.01.003

[11] May JR, Delves DM (1964) The survival of *H*. *influenzae* and pneumococci in specimen of sputum sent to the laboratory by post. J Clin Pathol 17: 254–256. 14159453 10.1136/jcp.17.3.254 PMC480734

[12] Monroe PW, Muchmore HG, Felton FG, Pirtle JK (1969) Quantitation of microorganisms in sputum. Appl Microbiol 18: 214–220. 4390055 10.1128/am.18.2.214-220.1969 PMC377946

[13] Jefferson HM, Dalton HP, Sacobar MR, Allison MJ (1975) Transportation delay and the microbiological quality of clinical specimens. Am J Clin Pathol 64: 689–694. 1190128 10.1093/ajcp/64.5.689

[14] Ross PW, Lough H (1978) Survival of upper respiratory tract bacteria on cotton wool swabs. J Clin Pathol 31: 430–433. 348727 10.1136/jcp.31.5.430 PMC1145297

[15] Perry JL (1997) Assessment of swab transport systems for aerobic and anaerobic organism recovery. J Clin Microbiol 35: 1269–1271. 9114423 10.1128/jcm.35.5.1269-1271.1997 PMC232745

[16] Ludwig W, Schleifer KH (1994) Bacterial phylogeny based on 16S and 23S rRNA sequence analysis. FEMS Microbiol Rev 15: 155–173. 7524576 10.1111/j.1574-6976.1994.tb00132.x

[17] Klouche M, Schröder U (2008) Rapid methods for diagnosis of blood-stream infections. Clin Chem Lab Med 46: 888–908. 10.1515/CCLM.2008.157 18624614

[18] Fenollar F, Raoult D (2007) Molecular diagnosis of bloodstream infections caused by non-cultivable bacteria. Int J Antimicrob Agents 30S: S7–S15. 10.1016/j.ijantimicag.2007.06.024 17707613

[19] Anrade SS, Bispo PJM, Gales AC (2008) Advances in the microbiological diagnosis of sepsis. Shock 30: 41–46. 10.1097/SHK.0b013e3181819f6c 18704010

[20] Jenkins C, Ling CL, Ciesielczuk HL, Lockwood J, Hopkins S, McHugh TD, et al (2012) Detection and identification of bacteria in clinical samples by 16S rRNA gene sequencing: comparison of two different approaches in clinical practice. J Med Microbiol 61: 483–488. 10.1099/jmm.0.030387-0 22160310

[21] Marks F, Adu-Sarkodie Y, Hünger F, Sarpong N, Ekuban S, Agyekum A, et al (2010) Typhoid fever among children, Ghana. Emerg Infect Dis 16: 1796–1797. 10.3201/eid1611.100388 21029549 PMC3294512

[22] Schwarz NG, Sarpong N, Hünger F, Marks F, Acquah SE, Agyekum A, et al (2010) Systemic bacteraemia in children presenting with clinical pneumonia and the impact of non-typhoid salmonella (NTS). BMC Infect Dis 10, 319 10.1186/1471-2334-10-319 21050455 PMC2991321

[23] Richter SS, Beekmann SE, Croco JL, Diekema DJ, Koontz FP, Pfaller MA, et al Minimizing the workup of blood culture contaminants: implementation and evaluation of a laboratory-based algorithm. J Clin Microbiol 40: 2437–2444. 12089259 10.1128/JCM.40.7.2437-2444.2002 PMC120579

[24] Christner M, Rohde H, Wolters M, Sobottka I, Wegscheider K, Aepfelbacher M (2010) Rapid identification of bacteria from positive blood culture bottles by use of matrix-assisted laser desorption-ionization time of flight mass spectrometry fingerprinting. J Clin Microbiol 48: 1584–1591. 10.1128/JCM.01831-09 20237093 PMC2863888

[25] Cilia V, Lafay B, Christen R (1996) Sequence heterogeneities among 16S ribosomal RNA sequences, and their effect on phylogenetic analyses at the species level. Mol Biol Evol 13: 451–461. 8742634 10.1093/oxfordjournals.molbev.a025606

[26] Hagen RM, Frickmann H, Elschner M, Melzer F, Neubauer H, Gauthier YP, et al (2011) Rapid identification of *Burkholderia pseudomallei* and *Burkholderia mallei* by fluorescence in situ hybridization (FISH) from culture and paraffin-embedded tissue samples. Int J Med Microbiol 301: 585–590. 10.1016/j.ijmm.2011.04.017 21658996

[27] Rosey AL, Abachin E, Quesnes G, Cadilhac C, Pejin Z, Glorion C, et al Development of a broad-range 16S rDNA real-time PCR for the diagnosis of septic arthritis in children. J Microbiol Meth 68: 88–93. 10.1016/j.mimet.2006.06.010 16904782

[28] Kawamura Y, Whiley RA, Shu SE, Ezaki T, Hardie JM (1999) Genetic approaches to the identification of the mitis group within the genus *Streptococcus* . Microbiology 45(Pt 9): 2605–2613. 10.1099/00221287-145-9-2605 10517614

[29] Clinical and Laboratory Standards Institute (2009) Interpretive criteria for identification of bacteria and fungi by DNA target sequencing. Approved standard MM18-A, 1st ed. Wayne, PA: Clinical and Laboratory Standards Institute. pp. 30–34.

[30] Bernasconi E, Valsangiacomo C, Peduzzi R, Carota A, Moccetti T, Funke G (2004) *Arthrobacter woluwensis* subacute infective endocarditis: case report and review of the literature. Clin Infect Dis 38: e27–e31. 14765360 10.1086/381436

[31] Junier P, Kim OS, Hadas O, Imhoff JF, Witzel KP (2008) Evaluation of PCR primer selectivity and phylogenetic specificity by using amplification of 16S rRNA genes from betaproteobacterial ammonia-oxidizing bacteria in environmental samples. Appl Environ Microbiol 74: 5231–5236. 10.1128/AEM.00288-08 18567688 PMC2519257

[32] Frickmann H, Loderstaedt U, Racz P, Tenner-Racz K, Eggert P, Haeupler A, et al (2015) Detection of tropical fungi in formalin-fixed, paraffin-embedded tissue: still an indication for microscopy in times of sequence-based diagnosis? Biomed Res Int 2015: 938721 10.1155/2015/938721 25961048 PMC4417575

[33] Flot JF (2007) CHAMPURU 1.0: a computer software for unraveling mixtures of two DNA sequences of unequal lengths. Mol Ecol Notes 7: 974–977.

[34] Commey JO, Rodrigues OP, Akita FA, Newman M (1994) Bacterial meningitis in children in southern Ghana. East Afr Med J 71: 113–117. 7925039

[35] Doughari HJ, Ndakidemi PA, Human IS, Benade S (2011) The ecology, biology and pathogenesis of *Acinetobacter* spp.: an overview. Microbes Environ 26: 101–112. 21502736 10.1264/jsme2.me10179

[36] Al-Khoja MS, Darrell JH (1979) The skin as the source of *Acinetobacter* and *Moraxella* species occurring in blood cultures. J Clin Pathol 32: 497–499. 469007 10.1136/jcp.32.5.497 PMC1145714

[37] Lefèvre PC, Blancou J, Dedieu L, Diallo A, Libeau G, Thiaucourt F. (1993) Field diagnostic kits: a solution for developing countries? Rev Sci Tech 12: 451–460. 8400385 10.20506/rst.12.2.690

[38] Mühl H, Kochem AJ, Disqué C, Sakka SG (2010) Activity and DNA contamination of commercial polymerase chain reaction reagents for the universal 16S rDNA real-time polymerase chain reaction detection of bacterial pathogens in blood. Diagn Microbiol Infect Dis 66: 41–49. 10.1016/j.diagmicrobio.2008.07.011 18722072

[39] Linow M (2012) Mastermix 16S –ultra sensitive detection of microbial DNA. Res Mol Microbiol 2: 1–2.

[40] March-Rosselló GA, Muñoz-Moreno MF, García-Loygorri-Jordán de Urriés MC, Bratos-Pérez MA (2013) A differential centrifugation protocol and validation criterion for enhancing mass spectrometry (MALDI-TOF) results in microbial identification using blood culture growth bottles. Eur J Clin Microbiol Infect Dis 32: 699–704. 10.1007/s10096-012-1797-1 23274860

[41] Frickmann H, Masanta WO, Zautner AE (2014) Emerging rapid resistance testing methods for clinical microbiology laboratories and their potential impact on patient management. Biomed Res Int 2014: 375681 10.1155/2014/375681 25343142 PMC4197867

