# Supplementary figures and images for: 16S rRNA Gene Sequence-Based Identification of Bacteria in Automatically Incubated Blood Culture Materials from Tropical Sub-Saharan Africa
Source: PLoS One. 2015 Aug 13;10(8):e0135923. doi: 10.1371/journal.pone.0135923 (PMC4535881; doi:10.1371/journal.pone.0135923)

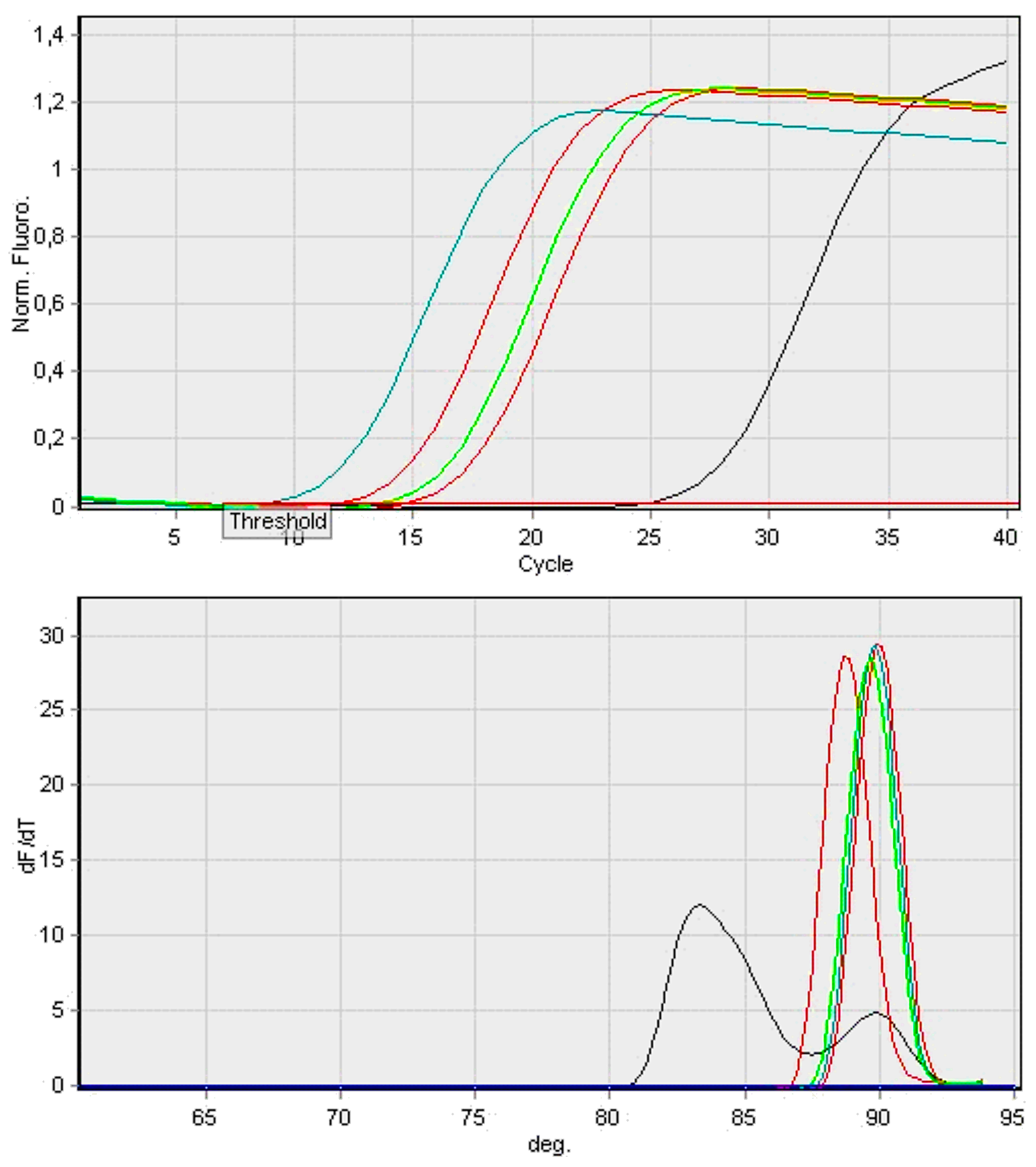

Supplement: S1 Fig — Amplification is depicted in the upper panel, melting curve analysis in the lower panel. The atypical melting peak in the negative sample (black curve) can be discriminated from the specific peaks of the positive samples (colored curves). (TIF) [file pone.0135923.s001.tif]
