# Supplementary material for: 16S rRNA Gene Sequence-Based Identification of Bacteria in Automatically Incubated Blood Culture Materials from Tropical Sub-Saharan Africa
Source: PLoS One. 2015 Aug 13;10(8):e0135923. doi: 10.1371/journal.pone.0135923 (PMC4535881; doi:10.1371/journal.pone.0135923)
Supplement: S1 Table — (DOC) [file pone.0135923.s002.doc]

**S1 Table. Comparison of Ct-values in PCR-positive samples with and without cultural growth.** No direct comparison of the two PCRs is intended.

| Diagnostic approach | 917 bp 16S rRNA gene PCR | | |
| --- | --- | --- | --- |
|  | Mean Ct-value (± standard deviation SD) for culture-positive, PCR-positive samples | Mean Ct-value (± SD) for culture-negative, PCR-positive samples | Two-tailed P-value (Mann–Whitney test) |
|  | 15.3 (±4.6) | 17.0 (±6.1) | P=0.0145 (significant) |
|  | Mean Ct-value (± SD) for detections of potentially etiologically relevant pathogens in culture-negative, PCR-positive samples | Mean Ct-value (± SD) for detections of presumed technical contaminations in culture-negative, PCR-positive samples | Two-tailed P-value (Mann–Whitney test) |
|  | 12.5 (±5.2) | 21.8 (±2.7) | P<0.0001 (significant) |
| Diagnostic approach | 357 bp 16S rRNA gene PCR | | |
|  | Mean Ct-value (± SD) for culture-positive, PCR-positive samples | Mean Ct-value (± SD) for culture-negative, PCR-positive samples | Two-tailed P-value (Mann–Whitney test) |
|  | 21.5 (±3.7) | 23.6 (±1.7) | P<0.0001 (significant) |
|  | Mean Ct-value (± SD) for detections of potentially etiologically relevant pathogens in culture-negative, PCR-positive samples | Mean Ct-value (± SD) for detections of presumed technical contaminations in culture-negative, PCR-positive samples | Two-tailed P-value (Mann–Whitney test) |
|  | 24.3 (±2.6) | 23.5 (±1.3) | P<0.0001 (significant) |
